# Antimicrobial stewardship in primary health care programs in humanitarian settings: the time to act is now

**DOI:** 10.1186/s13756-023-01301-4

**Published:** 2023-09-04

**Authors:** Claudia Truppa, Beatriz Alonso, Kate Clezy, Carole Deglise, Carole Dromer, Silvia Garelli, Carolina Jimenez, Rupa Kanapathipillai, Mohamad Khalife, Ernestina Repetto

**Affiliations:** 1 International Committee of the Red Cross, Geneva, Switzerland; 2 CRIMEDIM Center for Research and Training in Disaster Medicine, Humanitarian Aid and Global Health, University of Eastern Piedmont, Novara, Italy; 3 Médecins Sans Frontières, Barcelona, Spain; 4 Médecins Sans Frontières, Amsterdam, The Netherlands; 5 Médecins Sans Frontières, Geneva, Switzerland; 6 Médecins Sans Frontières, Paris, France; 7 Médecins Sans Frontières, Brussels, Belgium; 8 Service des Maladies Infectieuses, Clinique Hospitalière Universitaire Saint Pierre, Bruxelles, Belgium

**Keywords:** Antimicrobial resistance, Antimicrobial stewardship, Conflict, Humanitarian health

## Abstract

Fragile and conflict-affected settings bear a disproportionate burden of antimicrobial resistance, due to the compounding effects of weak health policies, disrupted medical supply chains, and lack of knowledge and awareness about antibiotic stewardship both among health care providers and health service users. Until now, humanitarian organizations intervening in these contexts have confronted the threat of complex multidrug resistant infections mainly in their surgical projects at the secondary and tertiary levels of care, but there has been limited focus on ensuring the implementation of adequate antimicrobial stewardship in primary health care, which is known to be setting where the highest proportion of antibiotics are prescribed. In this paper, we present the experience of two humanitarian organizations, Médecins sans Frontières and the International Committee of the Red Cross, in responding to antimicrobial resistance in their medical interventions, and we draw from their experience to formulate practical recommendations to include antimicrobial stewardship among the standards of primary health care service delivery in conflict settings. We believe that expanding the focus of humanitarian interventions in unstable and fragile contexts to include antimicrobial stewardship in primary care will strengthen the global response to antimicrobial resistance and will decrease its burden where it is posing the highest toll in terms of mortality.

## Background

The recent publication in the Lancet of the global estimates for the burden of antimicrobial resistance (AMR) has generated an earthquake in the public health community, showing robust evidence on the toll of AMR on global mortality. Such toll has been demonstrated to be disproportionately higher on low-income settings, particularly in Sub-Saharan Africa [1].

Low- and middle-income countries (LMIC) often bear an uneven burden of conflict, social or political violence, and institutional fragility, which add inevitably to the complexities of ensuring adequate surveillance, monitoring, and design of evidence-based interventions to tackle a variety of health issues, including AMR [2, 3].

Fragile and conflict affected settings often present the intrinsic challenge of dysfunctional health systems [4], and rely on external support, with a variety of humanitarian and development agents playing a complementary—and at times partly substitutive—role to that of local institutions in analysing the health situations; designing, implementing, and monitoring health interventions; and generating evidence to inform health programming [5, 6].

Over the last decade, there has been a growing interest among the global health community to better document effective and equitable approaches to deliver health care in fragile and conflict-affected settings [7–9]: from child health and nutrition interventions [10] to sexual and reproductive health care services [11]; from communicable disease control programs [12, 13] to non-communicable disease (NCD) care [14]. In such a landscape of progressive integration of approaches to strengthen existing health systems [15, 16], AMR has also become a critical element advocated for inclusion in equitable health policies for LMICs [17]. However, this seems to have received little to no attention so far in conflict situations.

In this comment, we would like to shed light on what we perceive as a neglected topic in humanitarian health, presenting the experience of two humanitarian organizations, and drawing lessons from both their programs and the available literature.

## Conflict and antimicrobial resistance: what do we know

In conflict-affected settings, several compounding factors can contribute to the emergence and spread of AMR [18, 19]. In addition to that, the implementation of adequate infection prevention and control (IPC) and antimicrobial stewardship (AMS) interventions have specific challenges, including, but not limited to: constrained access to quality water as key element for proper hygiene and sanitation in communities and health facilities; self-medication practices in countries with absence of law enforcement on sales of over-the-counter antibiotics; lack of awareness on the topic among health workers involved in service delivery; absence of laboratory infrastructure; and conflicting agendas in prioritization of programs when immediate life-saving priorities need to be addressed with limited resources [20–23].

Outpatient settings in high income countries (HIC) are known to be the level of care where most antibiotics are prescribed, and often inappropriately [24]. A recent literature review on antibiotic prescription practices in LMIC and primary health care (PHC) highlighted that antibiotics are highly prescribed also in these settings, often exceeding 50% of overall medical consultations, with a high proportion of inappropriate use [25]. The analysis performed did not take into consideration settings affected by acute conflicts or suffering from post-conflict situation, where the patterns of antibiotic prescriptions are not known and arguably as equal as, or worse than, the ones reported in the review.

In addition to that, the COVID-19 pandemic has contributed to a documented increase in inappropriate antibiotic prescriptions, which in turn can further aggravate the pre-existing burden of AMR [26, 27].

AMS has been extensively documented as a successful strategy to improve antibiotic prescription practices both in HIC  and in LMICs [28, 29]. However, it remains difficult for physicians and other health care workers to optimize antibiotic prescriptions in situations of perceived urgency, such as in intensive care units (ICUs) [30], or perceived uncertainty, such as for primary health care provision in remote areas, where it could be challenging to follow up closely on the clinical evolution of a potentially severe bacterial infection [31].

To address these difficulties and provide health care workers with evidence-informed guidance on antibiotic prescriptions, particularly in LMICs, the World Health Organization (WHO) has introduced since 2017 the Access, Watch and Reserve (AWaRe) classification for antibiotics in its essential medicine list, a tool to prioritize specific categories as targets of AMS intervention and monitoring [32]. More recently, the “WHO Essential Medicines List Antibiotic Book” initiative has suggested key implementation strategies to improve AMS at all levels of care, as a key component of quality of care [33]. However, the available toolkits for implementing AMS in resource limited settings, provided by both WHO and the Centers for Disease Control and Prevention (CDC), do not provide guidance specific to primary care facilities, remaining focused on hospitals [34, 35]. In these documents, moreover, there is no specific consideration for inclusion of AMS-inclusive preparedness measures related to situations in which priorities are competing and availabilities of diagnostics, human resources and medicines can be heavily affected, such as in acute humanitarian emergencies. In these situations, prescribing physicians can often be confronted with ethical dilemmas on how to balance patient-centred care with a public health approach focused on the broader population [36, 37], similarly to what is described in ICUs or remote areas in HICs [30, 31]. Humanitarian agencies, on the other hand, can also find themselves in the difficult position of having to decide between rapidly filling immediate gaps in supply chains or adopting stricter approaches when performing donations of medical materials [38]. This can lead to potentially counterproductive long term consequences in the absence of AMS-sensitive policies: as an example, some health emergency kits used by international medical organizations contain antibiotics on the “watch” list [39], which need to be made available in complex situations such as conflicts, but without providing adapted recommendation on how to monitor their prescription.

Over the last few years, increasing evidence has emerged on the positive value of AMS interventions in secondary and tertiary care, mainly in hospital projects, in conflict-affected settings, such as the Middle East [18, 20, 21]. However, to the best of our knowledge, there is a profound gap in the documentation of AMS implementation in PHC in these contexts: in fact, a rapid PubMed search of index terms and free keywords for the concepts “antibiotic/antimicrobial stewardship” and “primary health care” and “conflict”, retrieved zero results.

Considering the increasing evidence being produced on PHC interventions in conflict settings, we argue that there are already low hanging fruits that humanitarian agencies could make use of, integrating them with an AMS lens into their programs and policies. Below we share our experience on some of these, along with the existing evidence from LMICs and HICs that can reinforce our suggestions.

## The experience of two humanitarian organizations with antimicrobial resistance in conflict-affected settings: the approaches adopted so far

Médecins Sans Frontières (MSF) and the International Committee of the Red Cross (ICRC) are two international humanitarian organizations supporting the delivery of quality health care in conflict-affected areas in the acute and post-acute phase of conflicts.

MSF intervenes in conflict-affected areas with a variety of activities that include, among others, primary to secondary health care, acute trauma care, pediatric and neonatal care, malnutrition, sexual reproductive health care and mental health care. Depending on the country’s infectious diseases epidemiology, ensuring access to PHC services can include diagnosis and management of vaccine preventable diseases, malaria, dengue, Human Immunodeficiency Virus (HIV) infection, and tuberculosis. Quality of care is a core strategic ambition for MSF, ensuring patients and population are partners in the response, while effectiveness and safety of the medical intervention are promoted at all levels.

The ICRC has a specific mandate to intervene in armed conflict governed by the Geneva Conventions, and implements a range of health activities spanning from primary health care—including in places of detention—to mental health and psychosocial support, from trauma first aid to prehospital emergency care, from hospital care to physical rehabilitation. Its health strategy is anchored in three key interconnected principles: people centeredness, continuum of care, and public health approach, aiming to ensure the best possible access to quality care to the largest number of people affected by conflict and other situation of violence [40].

These two organizations have been confronted with the challenge of AMR in the delivery of health care interventions in multiple conflict settings and have attempted to both document and respond to it in different types of projects.

MSF has developed a structured approach to implement AMS in hospitals, including those situated in areas affected by conflicts or suffering the consequences of a recent conflict [41]: the strategy adopted combines standardized antibiotic treatment guidelines and context adapted standard antibiotic forms; identification, training and mentoring of antibiotic stewardship focal point clinicians; adoption of restrictive to persuasive stewardship strategies and regular point prevalence surveys for antibiotic prescriptions; and, whenever possible, access to diagnostic facilities that include quality assured microbiology laboratories, also via innovative approaches such as modular microbiology laboratories (MiniLab) and applications improving antibiotic susceptibility results interpretations (Antibiogo) [42, 43]. Moreover, in the last years, the organization has invested in understanding the drivers for antibiotic prescription among health care workers and of antibiotic use among patients and communities, in order to contextualize the approaches to the local reality [44, 45].

However, antibiotic stewardship in PHC interventions has been very limited compared to the inpatients setting and, consequently, neither a specific PHC strategy nor specific PHC tools have been developed to date, despite the fact that PHC facilities are the places where the highest amount of antibiotics are prescribed (more than 20 million of oral formulations of antibiotics have been ordered by MSF in 2020: internal data) and the quality of antibiotic prescriptions is still sub-optimal. For example, with no specific AMS intervention, the difference between the proportion of consultations that need an antibiotic prescription based on the diagnosis, and the proportion of consultation that had an antibiotic prescription varies, depending on the type of project, from 5 to 40% (internal MSF data from antibiotic prescription surveys).

Investment on improving quality of medical consultations in PHC via an electronic Clinical Decision Support System (eCDSS: MSF E-care) has been made for children under five years of age, and a similar approach is under development for children under 2 months of age: where the tool has been piloted, an improvement in the quality of medical consultation together with a clear trend on significant reduction of antibiotic prescription, up to 50%, has been seen for the majority of the clinical indications (internal MSF data, Kenya). Still, these tools have not been prioritized nor adapted yet for rapid deployment in acute conflict zones, nor for populations other than the pediatric one [46, 47].

The ICRC response to the challenges of AMS in conflict settings has focused so far on its hospital program. An extensive qualitative study was conducted analyzing enablers and barriers to the implementation of adequate IPC measures in its hospital projects [20], which has set the ground for the definition of an evidence-based approach to strengthen IPC awareness, processes, and practices. The first AMS structured protocol was adopted in a reconstructive surgical project running in Lebanon from 2015 to 2021, with a dedicated IPC officer and standard operating procedures defined for antibiotic prescription and administration, and with implementation of a full package of AMS involving IPC, antibiotic use optimization, including guidance on prescription based on antibiotic susceptibility testing performed in high quality laboratory services [48]. 

As for MSF, there has been limited engagement on AMS at the primary level of care. However, with the implementation of ALMANACH (Algorithm for the Management of Childhood illness), a digital clinical decision support tool first implemented in Nigeria, an improvement in prescription practices at the primary level of care was documented, including a reduction in the use of antibiotics, which triggered the interest for the potential impact the scale up of these tools could have in addressing the growing threat of AMR in low resourced settings [27–29].

The promising results on the effectiveness of electronic clinical decision support algorithms on antibiotic prescription practices need however to be corroborated by further studies in other settings, to understand how generalizable these are. Similar approaches have been documented in low- and middle-income countries [52], but evidence from conflict-affected settings is still limited.

## Humanitarian primary health care interventions in conflict-affected settings: what can we do better

Despite the increased risk for AMR in conflict zones, and the work done by MSF and the ICRC in hospital projects in these settings, a clear gap in evidence on AMS at the PHC specifically in conflict setting persists, as well as a lack of documentation of good clinical practices in this regard, if any are piloted. Here we provide eight key recommendations that in our opinion can represent first steps in the right direction.

First of all, there is a need to provide a guiding framework to all humanitarian actors in order to streamline appropriate antibiotic prescription in their PHC interventions in crises settings: to this end, a specific inter-agency working group could be established, and specific quality standards of AMS included within the Sphere standards, on the basis of the WHO AWaRe classification of antibiotics taking into account the experience of local health organization as well as international health organization.

Second, specific evidence-based recommendations need to be updated where existing, and formulated were absent, with regards to antibiotic treatment protocols. For example, the systematic use of empirical broad-spectrum antibiotics for the treatment of severe acute malnutrition has also been challenged, but with no conclusive evidence on the possibility to suspend this practice has been collected [28]. At the same time, specific focus could be dedicated in better understanding antibiotic prescription practices in some key PHC services where antibiotics are known to be sub-optimally prescribed in HIC—such as in pregnancy  [54] or chronic obstructive pulmonary disease (COPD) exacerbations in adults [55].

Third, we have already described how clinical decision support electronic algorithms have the potential of offering a powerful tool to improve prescription practices, particularly in situations in which there is critical lack of skilled health care workforce [46, 47, 49, 50, 52]. Their use could be promoted and scaled up in humanitarian interventions, and they could be customized on the local culture and epidemiology, which entails the need to secure funding for their maintenance and regular updates.

Fourth, specific contextualized AMS training packages need to be elaborated, and innovative ways of ensuring continuous capacity building could be developed, such as decentralized learning and mentoring strategies, training of trainers, establishment of communities of practice, among other options.

Fifth, empowering all health care workers to be active in AMS, particularly in contexts with a depleted health care workforce: in particular, the role of nurses, social workers, infection control officers, health promoters, and pharmacists in AMS could be promoted, as task shifting and task sharing have been proven as successful strategies for resource-limited settings in many health domains, and particularly in NCD management [56–58].

Sixth, more investment in qualitative and health system level research is needed, to appreciate the complexity of the multidisciplinary approach needed in AMS interventions [59]. Such approach would allow to better document the root causes of inappropriate antibiotic prescription and consumption in humanitarian settings, in order to design AMS strategies tailored to the specificities of these contexts.

Seventh, based on the context-specific evidence documented, meaningful strategies of community engagement and capacity building of frontline and lay health care workers need to be developed, as well as practical tools promoting behavioural shifts both in antibiotic prescription and consumption [60].

Finally, the development of AMR National Action Plans where absent, or their regular updates where present, needs to be supported, and strengthened through the inclusion of specific contingency plans for countries characterized by socio-political fragility which are known to harbour a high burden of AMR: this would allow to have a solid base of evidence to strengthen AMS at all levels of care and interventions promoted by both national governments and the international community, in case a sudden wave of instability and violence arises. The recent onset of the conflict in Ukraine provides the perfect, unfortunate example of how the disruption generated by a war can exacerbate the pre-existing threats of AMR [61, 62].

## Conclusion

If we agree that a global response to the increasing threat of antibiotic resistance is needed, we need to acknowledge that such response cannot be complete, let alone successful, without addressing the need for AMS in primary care settings also in countries affected by fragility and conflict [63]. More can be done: the time to act is now.

## Data Availability

Not applicable.

## Abbreviations

ALMANACH: Algorithm for the Management of Childhood Illness
AMR: Antimicrobial resistance
AMS: Antimicrobial stewardship
AWaRe: Access, Watch and Reserve
CDC: Centers for Disease Control and Prevention
COPD: Chronic obstructive pulmonary disease
HIC: High Income Countries
eCDSS: Electronic Clinical Decision Support System
HIV: Human immunodeficiency virus
ICRC: International Committee of the Red Cross
ICU: Intensive Care Unit
IPC: Infection Prevention and Control
LMIC: Low- and Middle-Income Countries
MSF: Médecins sans Frontières
NCD: Non-communicable disease
PHC: Primary Health Care
WHO: World Health Organization
